# Alphaherpesvirus Subversion of Stress-Induced Translational Arrest

**DOI:** 10.3390/v8030081

**Published:** 2016-03-15

**Authors:** Renée L. Finnen, Bruce W. Banfield

**Affiliations:** Department of Biomedical and Molecular Sciences, Queen’s University, Kingston, ON K7L 3N6, Canada; renee.finnen@queensu.ca

**Keywords:** alphaherpesvirus, translational arrest, stress granules

## Abstract

In this article, we provide an overview of translational arrest in eukaryotic cells in response to stress and the tactics used specifically by alphaherpesviruses to overcome translational arrest. One consequence of translational arrest is the formation of cytoplasmic compartments called stress granules (SGs). Many viruses target SGs for disruption and/or modification, including the alphaherpesvirus herpes simplex virus type 2 (HSV-2). Recently, it was discovered that HSV-2 disrupts SG formation early after infection via virion host shutoff protein (vhs), an endoribonuclease that is packaged within the HSV-2 virion. We review this discovery and discuss the insights it has provided into SG biology as well as its potential significance in HSV-2 infection. A model for vhs-mediated disruption of SG formation is presented.

## 1. Translational Arrest in Response to Stress

Cellular responses to stressful stimuli can be broadly categorized as destructive, resulting in cell death or protective, resulting in cell survival. The general shutdown and reprogramming of translation that occurs in cells in response to stress can be considered protective: by stopping the synthesis of most proteins, cellular resources are thereby conserved and can, instead, be focused on synthesizing those proteins required for surviving the stress.

Regulation of cap-dependent translation occurs primarily at the level of initiation, though recent evidence suggests that regulation can also occur at the level of elongation [1]. Assembly of both of the macromolecular complexes required for translation initiation, the 43S ribosomal pre-initiation complex (43S PIC) and the eIF4F cap-binding complex, can be targeted to shut down translation. The 43S PIC is targeted through the activation of eukaryotic initiation factor 2 (eIF2) kinases. Eukaryotic cells employ four eIF2 kinases to sense stressful conditions in their environment [2]. The stressors that eIF2 kinases can sense include environmental changes like elevated temperature (heat shock), accumulation of misfolded proteins, starvation and oxidation. In addition to sensing environmental stress, the double-stranded RNA (dsRNA)-dependent protein kinase (PKR) can sense the presence of viruses. Following the sensing of environmental stress or viral infection, eIF2 kinases become activated and phosphorylate the alpha subunit of eIF2 (eIF2α), ultimately leading to a failure to assemble the 43S PIC [3,4,5,6]. Many different stressors result in reduced activity of the mechanistic target of rapamycin (mTOR) kinase. Two components of eIF4F, the cap-binding protein eIF4E and the RNA helicase eIF4A, are affected by independent mechanisms stemming from reduced activity of the mTOR complex 1 (mTORC1), resulting in reduced assembly and functionality of eIF4F [3,4,5,6].

Subsets of cellular mRNAs continue to be selectively translated when cap-dependent translation is shut down. Many of these mRNAs encode stress response proteins that facilitate survival and recovery such as chaperones [7,8], repair enzymes [9], and apoptosis inhibitors [10,11,12] as well as transcriptional activators of stress response genes [13,14]. Initiation of translation on these mRNAs proceeds by cap-independent mechanisms. The best-characterized of these mechanisms is the use of internal ribosome entry sites (IRESs), complex structural elements found in the 5′ untranslated regions (5′ UTRs) of mRNAs. First identified in poliovirus [15,16], IRESs directly recruit ribosomes without the requirement for some or even all initiation factors [17,18]. With the advent of recent technologies such as ribosome profiling [19], we stand poised to discover more details about these cap-independent translation mechanisms as well as the breadth of translational reprogramming in eukaryotic cells during times of stress.

## 2. Alphaherpesvirus Subversion of Translational Arrest

Stoppages in protein synthesis are problematic for all viruses because they rely on the host cell’s translation machinery for the production of their proteins. Thus viruses must counteract the strategies used by cells to inhibit translation initiation or employ non-canonical modes of translation initiation to facilitate continued protein synthesis and promote their replication. The wide variety of tactics used by viruses to ensure synthesis of their proteins has been comprehensively reviewed by Walsh *et al.* [18]. The tactics specifically used by the alphaherpesviruses, the main subject of this article, have primarily been studied in herpes simplex virus type 1 (HSV-1). HSV-1 utilizes four proteins to counteract activation of eIF2 kinases and the resulting phosphorylation of eIF2α: Us11 blocks PKR activation by binding dsRNA [20,21]; vhs blocks PKR activation via its endoribonuclease activity [22]; glycoprotein B (gB) blocks the ability of the PKR related endoplasmic reticulum kinase (PERK) to sense protein misfolding in the endoplasmic reticulum by binding the luminal domain of PERK [23]; and ICP34.5 recruits cellular protein phosphatase 1a to dephosphorylate eIF2α [24]. These viral proteins carry out their antagonistic roles at different times during infection from the immediate onset of viral infection (vhs) to early after viral DNA synthesis (ICP34.5) to late in the infection (gB and Us11) allowing HSV-1 to continuously counteract eIF2 kinase activation [22]. Other alphaherpesviruses, such as varicella zoster virus (VZV) and pseudorabies virus (PRV), do not encode homologues of Us11 or ICP34.5 and use additional viral proteins to prevent phosphorylation of eIF2α. The VZV virion component ORF63 and the PRV immediate early protein IE180 have both been implicated in the suppression of eIF2α phosphorylation [25,26]. To ensure the assembly of eIF4F, the HSV-1 serine/threonine kinase, Us3, promotes the constitutive activation of mTORC1 [27], the immediate early protein, ICP0, promotes the incorporation of eIF4E into eIF4F [28] and the chaperone-like activity of ICP6 promotes the interaction of eIF4F components eIF4E and eIF4G [29]. In addition to these counteractive tactics, non-canonical mechanisms are used for the translation of some alphaherpesvirus mRNAs. IRES-mediated translation has been described for HSV-1 thymidine kinase [30] and for Marek’s disease virus RLORF9 protein [31,32,33]. Although vhs is most often described as an endoribonuclease, there is evidence that it can also play a role as a translational modulator [34,35]. In this role, vhs can enhance cap-independent translation of mRNAs via *cis*-acting elements in their 5’ UTRs including cellular mRNAs known to contain an IRES as well as viral mRNAs encoding thymidine kinase, UL12 and gC [35]. Moreover, the ability of some of these *cis-*acting elements to direct translation can be strictly vhs-dependent.

## 3. Stress Granule Formation as a Consequence of Translational Arrest

A consequence of stoppages in protein synthesis is the formation of non-membrane bound cytoplasmic compartments known as stress granules (SGs). SGs are primarily composed of translationally silent mRNAs that remain associated with a cadre of translation initiation proteins, poly(A)-binding protein (PABP) and the small ribosomal subunit in the form of messenger ribonucleoprotein complexes (mRNPs) [36,37,38]. SGs can be quite irregular in shape and variable in size, ranging from 0.1 to 2 µm in diameter [39]. While their protein composition can also be variable, core mRNP components serve as definitive markers of SGs and certain cellular mRNA binding proteins, such as Ras-GTPase-activating SH3-domain-binding proteins (G3BP-1 and G3BP-2) and T cell internal antigen 1 (TIA-1), can also serve as reliable markers of these compartments [37].

SGs form rapidly in response to stress-induced phosphorylation of eIF2α or in response to compounds that stop protein synthesis by means that are mechanistically distinct from eIF2α phosphorylation, such as puromycin and pateamine A (PatA) [40,41,42]. Conversely, compounds that keep mRNAs associated with 80S ribosomes, such as cycloheximide and emetine, discourage SG formation [42]. These observations have led to a SG assembly model whereby the sudden increase in cytoplasmic levels of free mRNPs that follows stoppages in protein synthesis is the seminal event that initiates SG assembly [37]. The requirement for RNA in SG assembly is supported by the recent studies of Pastré and colleagues, who demonstrated that delivery of exogenous single stranded nucleic acid (RNA or DNA) to cells could promote SG assembly [43], and by the recent *in vitro* studies of Cech and colleagues, who demonstrated that the formation of higher-order assemblages of the RNA binding protein Fused in Sarcoma (FUS) could be seeded by the addition of RNA [44].

The steps leading from an increase in cytoplasmic levels of free mRNPs through to the assembly of microscopically visible cytoplasmic granules are ill-defined. The involvement of cellular proteins with both RNA binding capacity and prion like domains, such as G3BP and TIA-1, in SG assembly is well established [45,46] implicating the importance of RNA-protein and protein-protein interactions in assembling these structures. For G3BP in particular, SG assembly is inhibited by cleavage of G3BP-1 [47] or by disruption of ability of either G3BP-1 or G3BP-2 to bind other SG proteins [48]. Many post-translational modifications to SG proteins have also been implicated in SG assembly including dephosphorylation [46], methylation [49,50], deacetylation [51], ubiquitination [51], *O*-GlcNac modification [52] and poly(ADP ribosyl)ation (PARylation) [53,54,55]. Weak intermolecular interactions facilitated by RNA binding proteins with prion like domains in combination with these various post-translational modifications has been proposed by Alberti and colleagues to allow mammalian SGs to exist in a liquid droplet-like state that facilitates trafficking of components into and out of SGs [56]. Finally, interactions between mRNPs and the cytoskeleton are also required for SG assembly [57,58,59,60,61]. The conventional model of SG assembly along with the physical state in which SGs exist have been challenged by very recent work published by Parker and colleagues [62]. Their analysis of SGs using super-resolution microscopy suggested that SGs have a stable core structure surrounded by a more dynamic shell. Moreover, their success in purifying and determining the protein composition of these SG cores has important implications for the field. Like SG assembly, SG disassembly can proceed rapidly and is mechanistically ill-defined. Reversal of some of the post-translational modifications implicated in SG assembly can cause SG disassembly. For example, phosphorylation of SG proteins G3BP [46], growth factor receptor-bound protein 7 (Grb7; [63]) and dual specificity tyrosine-phosphorylation-regulated kinase 3 (DYRK3; [64]) all lead to SG disassembly, as does removal of PAR modifications by PAR glycohydrolases [55].

The primary role postulated for SGs is for temporary storage of cellular mRNAs during periods of stress [37,65]. As these mRNAs remain associated with components of the translation machinery, protein synthesis can resume quickly once the stress subsides and SGs are disassembled, thus helping the cell survive the period of stress. However, recent studies by Huttelmaier and colleagues provide evidence that mRNA translation and stabilization is largely independent of SG formation [66], suggesting that SGs likely play additional roles to enable stress survival. One new role that is particularly pertinent to viral infection is to promote innate immune responses. Fujita and colleagues demonstrated that two cellular sensors of viral RNA that trigger innate immune responses, PKR and retinoic acid inducible gene I (RIG-I), both localize to SGs that are induced following viral infection [67,68]. Several proteins involved in regulating RIG-I also localize to SGs [69,70,71]. Furthermore, Lloyd and colleagues have described SG-specific activation of PKR. According to their model, SG proteins G3BP-1 and caprin-1 form a complex with inactive PKR in the cytoplasm and, following the induction of stress, this complex is recruited into SGs where PKR becomes activated in a manner that is independent of foreign dsRNA recognition [72,73]. Activated PKR is subsequently released from SGs where it can proceed to shut down translation as well as trigger other PKR-mediated responses [74,75,76,77]. These links between SG formation and innate immune responses have led to the proposal that SGs serve as platforms for recognizing and responding to viral infections [70], which may explain why so many viruses destroy or modify these cytoplasmic compartments during infection (for overviews of the impact of various viruses on SGs, see references [70,78,79,80,81]). By understanding how viruses manipulate SGs, we stand not only to learn more about virus/host interactions but also to gain insights into the incompletely understood cycle of SG assembly and disassembly. As aberrant SG formation is a feature associated with several neurodegenerative diseases in humans [82,83,84,85], these insights may prove valuable for understanding and treating these diseases.

## 4. HSV-2 Infection Differentially Impacts Stress Granule Formation

The importance of SG formation in suppressing HSV infection was suggested by the enhanced growth of HSV-1 in cells in which production of the SG component TIA-1 or the related protein, TIAR, had been knocked out [86]. Infection of cells by HSV activates at least two eIF2 kinases, and yet accumulation of SGs is not observed following infection by HSV-1 strains F and KOS [87,88] or by HSV-2 strains HG52, G and 186 [89,90]. While these observations may be reflective of the combined abilities of Us11, vhs, gB and ICP34.5 to counteract the activities of PKR and PERK, they may be indicative of a direct impact of HSV infection on SG formation. When we investigated the ability of cells infected with HSV-2 to form SGs following arsenite treatment to induce oxidative stress [91], we discovered that infected cells had a disruption in their ability to form SGs in response to the arsenite treatment [90]. HSV-2-infected cells maintained the ability to phosphorylate eIF2α in response to the arsenite treatment indicating that, mechanistically, this disruption occurs downstream of eIF2α phosphorylation. Consistent with a block to SG formation occurring irrespective of the phosphorylation status of eIF2α, we observed that HSV-2 infected cells also have a disruption in their ability to form SGs in response to PatA. PatA disrupts translation initiation in an eIF2α-independent manner by irreversibly binding to eIF4A and modulating its activity [5,40,41]. In our initial SG disruption analyses, we followed TIA-1 as a marker of SGs. In subsequent assays using antibodies against G3BP to follow SG formation, we observed that G3BP positive SGs remained in infected cells following PatA treatment, however, these remaining SGs were largely devoid of TIA-1 [90]. This contrasted the situation in infected cells treated with arsenite where SGs were not detectable using either marker.

The differential impact of HSV-2 infection on SGs induced by arsenite *versus* those induced by PatA may arise as a result of fundamental differences in the properties of SGs induced by different mechanisms. PatA-induced SGs can persist for as long as 12 h post-treatment [92] whereas arsenite-induced SGs are much more rapidly disassembled [38]. If PatA-induced SGs are inherently more stable than their arsenite-induced counterparts, their disassembly as a consequence of HSV-2 infection may proceed with different kinetics or with a different order of departure of SG components allowing G3BP positive, TIA-1 negative SGs to remain in infected cells following PatA treatment. Alternatively, if the requirement for TIA-1 in assembling arsenite-induced SGs is more stringent than for PatA-induced SGs, viral modulation of TIA-1 may result in greater inhibition of SG formation in response to arsenite treatment as compared to PatA treatment. We have, in fact, observed an effect on TIA-1 localization following HSV-2 infection. At late times post-infection, TIA-1 localizes to novel nuclear structures that contain the RNA binding protein 68-kDa Src-associated in mitosis (Sam68; [90]). Understanding the differential impact of HSV-2 infection on SG formation will hinge on establishing whether the disruption in SG formation observed in HSV-2 infected cells occurs as a result of promotion of SG disassembly or prevention of SG assembly or as a result of both of these activities. We noted in our initial studies on the impact of HSV-2 on SG formation that the number of cells containing SGs as a result of over expression of GFP-TIA-1 was noticeably diminished when these cells were subsequently infected [90]. These results imply that disassembly of preformed SGs may be enhanced in the presence of HSV-2. We are currently developing more rigorous assays for determining whether HSV-2 infection can promote the disruption of preformed SGs.

## 5. HSV-2 vhs Interferes with Stress Granule Formation

**Figure 1:**
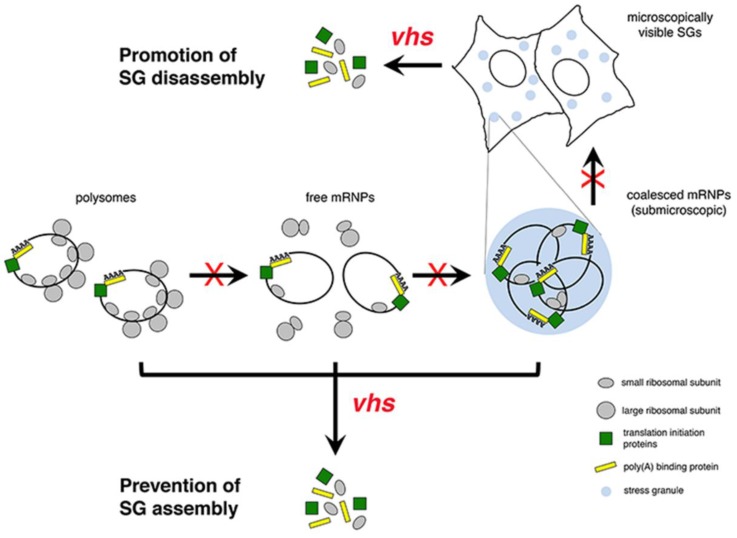
Model of virion host shutoff protein (vhs) endoribonuclease-mediated disruption of stress granule (SG) formation. Two different modes of disruption by vhs are proposed. vhs endoribonuclease activity promotes the destruction of mRNAs present in existing SGs leading to their disassembly. Alternatively or additionally, vhs endoribonuclease activity promotes the destruction of mRNAs present in polysomes, free messenger ribonucleoproteins (mRNPs) or submicroscopic coalesced mRNPs preventing the ensuing step in the SG assembly pathway. For simplicity, microscopically visible SGs as well as submicroscopic coalesced mRNPs that serve as SG nucleation sites are both represented as blue circles; microscopically visible SGs are often irregular in shape and are variable in size.

An obvious question stemming from our discovery that HSV-2 infection disrupted arsenite-induced SG formation was which viral protein(s) was responsible for this activity. We demonstrated that disruption of arsenite-induced SG formation in infected cells was detectable as early as 30 min post infection [89] and occurred in the absence of both viral DNA replication [90] and viral gene transcription [89]. In addition, disruption of arsenite-induced SG formation was not detected when virions were removed from the inoculum [90]. These observations indicated that this disruption could be mediated by a virion component. Two independent research groups had previously reported that spontaneous SGs form late in infection in cells infected with vhs-defective strains of HSV-1 [87,88]. This connection between vhs and SGs coupled with the well-established fact that vhs is a component of the alphaherpesvirus virion [93,94,95] made vhs a likely candidate to mediate this disruption. We demonstrated that cells infected with HSV-2 strains carrying defects in vhs were defective in their ability to disrupt arsenite-induced SG formation and that this ability was restored when a defect in vhs was specifically repaired [89]. Furthermore, disruption of arsenite-induced SG formation was observed in cells transfected with vhs expression constructs, indicating that vhs could mediate this disruption in the absence of other viral proteins.

vhs is an endoribonuclease [96,97,98,99] encoded by the UL41 gene [100,101]. Packaging of vhs into virions allows this endoribonuclease to be delivered to the cytoplasm immediately upon fusion of viral and cellular membranes and to commence the degradation of cellular mRNAs in advance of *de novo* viral gene expression. Cleavage of most mRNAs by vhs *in vivo* occurs within the 5′ UTR near the cap and the resulting decapped mRNAs are then rapidly degraded by the cellular 5′-3′ exonuclease Xrn1 [102,103]. Targeting of vhs endoribonuclease activity towards mRNAs *in vivo* may be facilitated by its ability to bind the cellular translation initiation factors eIF4A, eIF4B and eIF4H [104,105]. The ability of vhs to bind to translation initiation factors may also be indicative of a direct role for vhs in translation. This notion is supported by the studies of Smiley and colleagues who demonstrated that vhs activates cap-independent translation via *cis*-acting elements and also enhances the translation of viral late mRNAs [34,35,87,106]. As vhs may interact with mRNA in two distinct manners and has the ability to bind eIF4A, eIF4B, and eIF4H, all of which are known SG components [107], predicting which vhs activity is required to disrupt SG formation is not entirely straightforward. However, given that free mRNP is a key ingredient in SG assembly [43], the simplest model of how vhs disrupts SG formation, depicted in the lower portion of Figure 1, is that destruction of mRNA (as a component of polysomes, free mRNPs or submicroscopic coalesced mRNPs) by the combination of vhs endoribonuclease and Xrn1 exonuclease activities prevents SGs from assembling into microscopically visible structures. Alternatively or additionally, destruction of the mRNA contained within microscopically visible SGs by vhs and Xrn1 could drive the disassembly of these structures. In this mode, vhs can be considered a SG disaggregase. If vhs endoribonuclease activity is required for it to function as a SG disaggregase, it follows that maintaining intact mRNA is crucial for maintaining SG integrity. It also follows that both vhs and Xrn1 should be able to localize to SGs. We have observed localization of vhs at SGs induced in HeLa cells transfected with an endoribonuclease deficient vhs construct suggesting that vhs has this ability [108]. While Xrn1 is often described as a marker of processing bodies, another type of cytoplasmic RNA granule often found associated with SGs, small amounts of Xrn1 have been found to localize to SGs [109]. Our future studies will take advantage of the wealth of knowledge about functional domains and residues within vhs [105,110,111] to define which activities contribute to disruption of SG formation and to distinguish between the two possible modes of vhs-mediated disruption of SG formation depicted in Figure 1.

## 6. Significance of vhs-Mediated Disruption of Stress Granule Formation

The recent work connecting SGs with innate immune responses [67,70,72,73] has reinforced the view of SGs as antiviral in nature. We have demonstrated that vhs-mediated disruption of SG formation can be detected as early as 30 min after infection [89], a timeframe consistent with counteraction of an early antiviral response involving SGs. We propose that vhs-mediated promotion of SG disassembly and/or prevention of SG assembly counteracts SG-specific activation of innate immune responses. Lloyd and colleagues have speculated that SG-specific activation of PKR may arise from interplay between G3BP-1 and caprin-1 without requiring RNA ligands [72]. If SG-specific activation of PKR were truly independent of RNA ligands, vhs disruption of this mode of PKR activation would differ from the model of vhs-mediated PKR inactivation described by Roizman and colleagues. This model proposes that vhs-mediated inhibition of PKR arises as a result of clearance of self-annealing RNAs that carry double-stranded stretches capable of activating PKR [22]. Counteraction of SG-specific activation of innate immune responses may be an evasion strategy shared by other viruses that encode an endonuclease known to be involved in restricting host gene expression such as the gammaherpesviruses Kaposi’s sarcoma-associated herpesvirus (SOX; [112,113]), murine gammaherpesvirus 68 (muSOX; [114]), and Epstein-Barr virus (BGLF5; [115]) as well as influenza A virus (PA-X; [92,116]).
